# Supplementary figures and images for: Multiparameter immunoprofiling for the diagnosis and differentiation of progressive versus nonprogressive nontuberculous mycobacterial lung disease–A pilot study
Source: PLoS One. 2024 Apr 19;19(4):e0301659. doi: 10.1371/journal.pone.0301659 (PMC11029658; doi:10.1371/journal.pone.0301659)

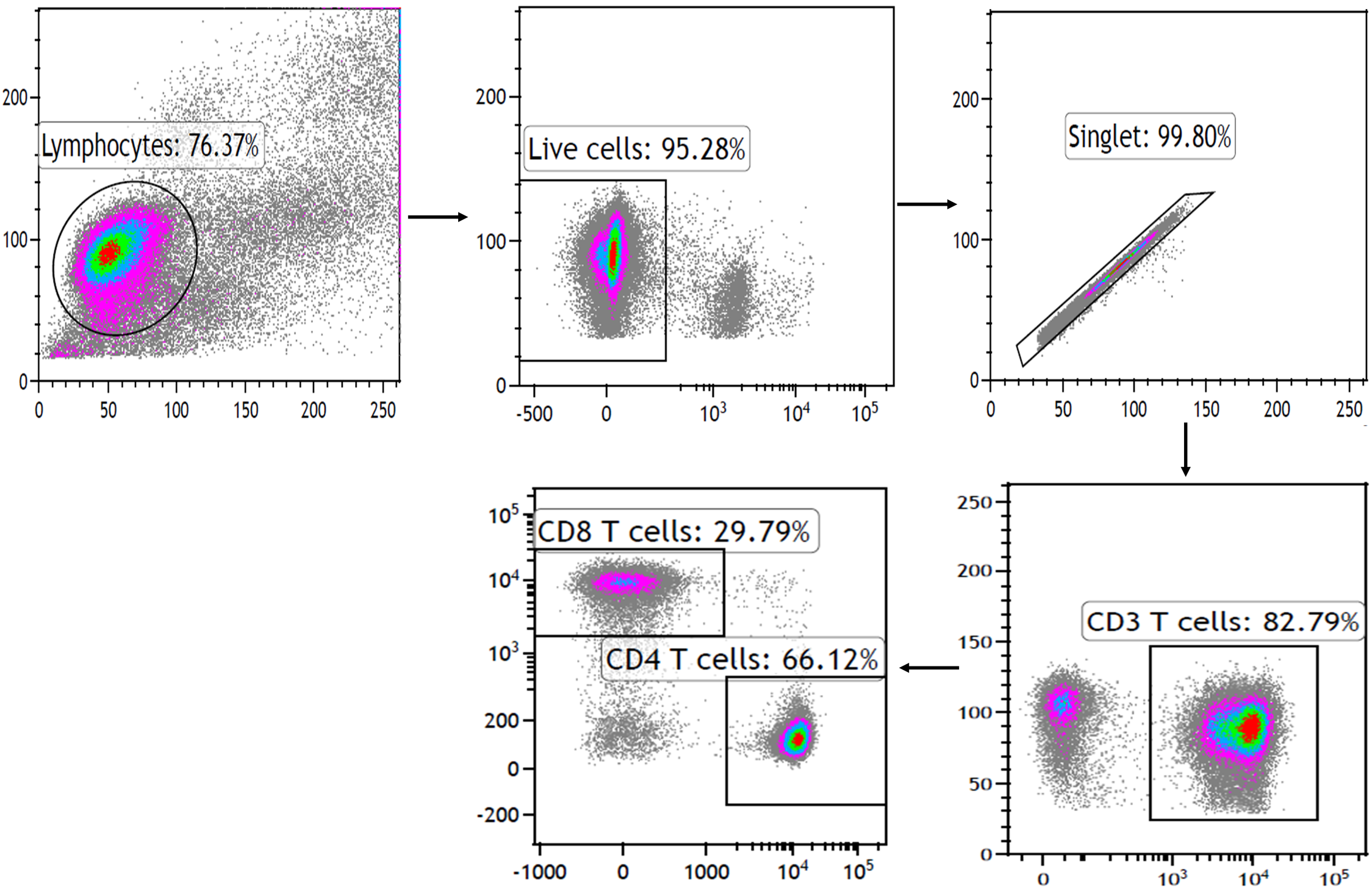

Supplement: S1 Fig — Representative gating strategy followed for gating CD3 from lymphocyte population. CD3 T cells gated in the live singlet gate of PBMC and subsequently CD4 and CD8 T cells were gated. (TIF) [file pone.0301659.s001.tif]

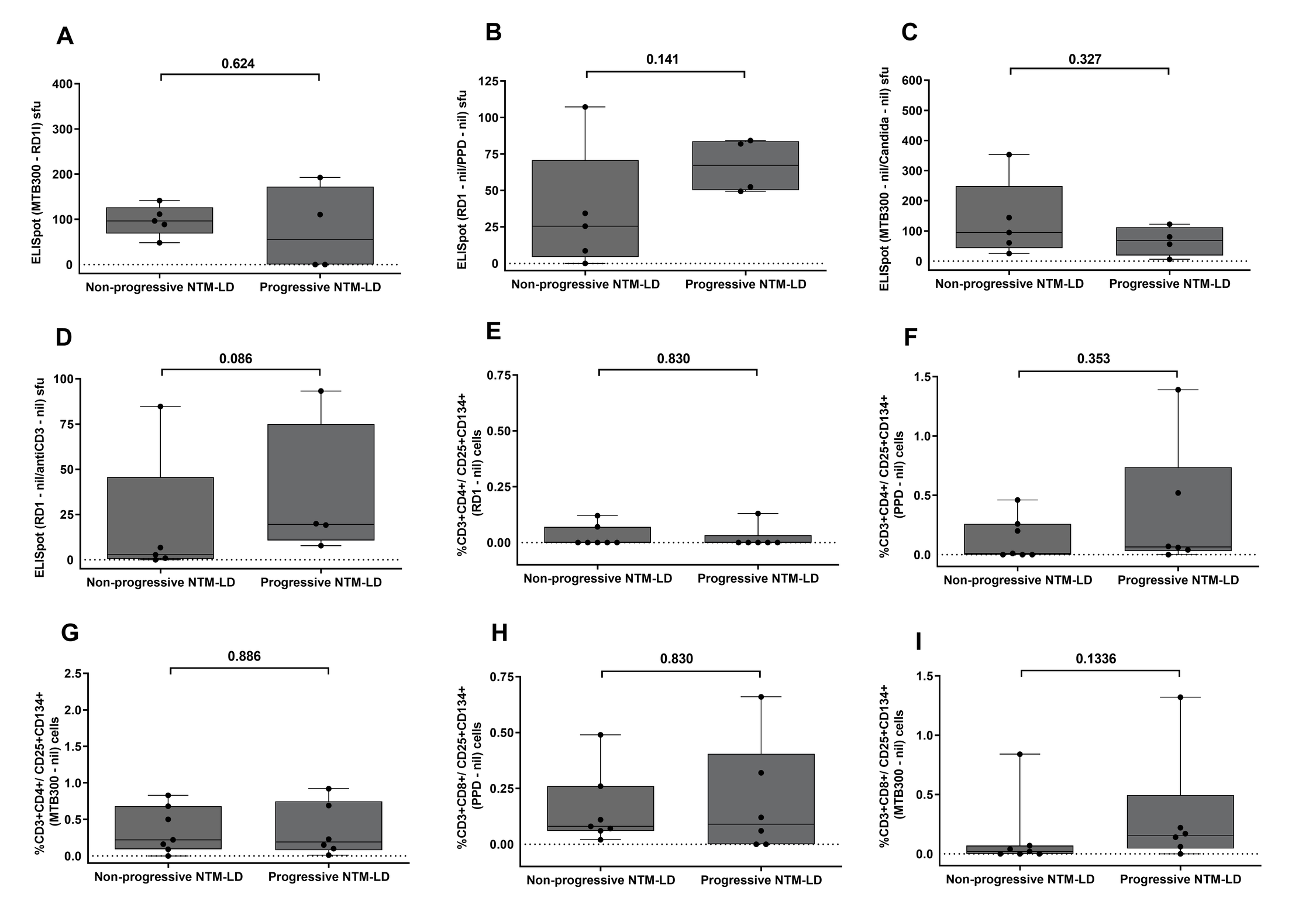

Supplement: S2 Fig — Testing results of IFN-γ ELISpot and flow cytometric CD4+/CD8+CD25+CD134+ T cells in nonprogressive and progressive MAC-LD: ELISpot results of MTB300-RD1 sfu (A), net ratios of RD1/PPD sfu (B), MTB300/Candida sfu (C) and RD1/antiCD3 sfu (D). Flow cytometric detection of percentage of CD3+CD4+CD25+CD134+ against RD1 peptides (E), PPD (F), MTB300 peptide pool (G). Percentage of CD3+CD8+CD25+134+ with PPD (H) and MTB300 peptide pool (I). The response by stimulated cells was background subtracted for each donor. Differences between the groups were compared using a Mann–Whitney U-test. The boxes show the median and interquartile range, and the whiskers show minimum and maximum values. (TIF) [file pone.0301659.s002.tif]
